# Programme science methodologies and practices that address “FURRIE” challenges: examples from the field

**DOI:** 10.1002/jia2.26283

**Published:** 2024-07-10

**Authors:** James R Hargreaves, Solange Baptiste, Parinita Bhattacharjee, Frances M Cowan, Michael E Herce, Krista Lauer, Izukanji Sikazwe, Elvin Geng

**Affiliations:** ^1^ Faculty of Public Health and Policy London School of Hygiene & Tropical Medicine London UK; ^2^ International Treatment Preparedness Coalition Johannesburg South Africa; ^3^ Institute for Global Public Health University of Manitoba Winnipeg Manitoba Canada; ^4^ Partners for Health and Development in Africa Nairobi Kenya; ^5^ CeSHHAR Harare Zimbabwe; ^6^ Liverpool School of Tropical Medicine Liverpool UK; ^7^ CIDRZ Lusaka Zambia; ^8^ Institute for Global Health and Infectious Diseases University of North Carolina Chapel Hill USA; ^9^ Division of Infectious Diseases and Center for Dissemination and Implementation Washington University in St. Lous St. Louis Missouri USA

**Keywords:** programme science, STI, HIV prevention, HIV care continuum, implementation, methodology

## Abstract

**Introduction:**

“Programme science” deploys scientific methods to address questions that are a priority to support the impact of public health programmes. As such, programme science responds to the challenges of making such studies: (1) feasible to undertake, (2) useful, (3) rigorous, (4) real‐world‐relevant, (5) informative, and undertaken by (6) equitable partnerships. The acronym “FURRIE” is proposed to describe this set of six challenges. This paper discusses selected HIV/STI (sexually transmitted infection) programme science case studies to illustrate how programme science rises to the FURRIE challenges.

**Discussion:**

One way in which programme science is made more feasible is through the analysis and interpretation of data collected through service delivery. For some questions, these data can be augmented through methods that reach potential clients of services who have not accessed services or been lost to follow‐up. Process evaluation can enhance the usefulness of programme science by studying implementation processes, programme−client interactions and contextual factors. Ensuring rigour by limiting bias and confounding in the real‐world context of programme science studies requires methodological innovation. Striving for scientific rigour can also have the unintended consequence of creating a gap between what happens in a study, and what happens in the “real‐world.” Community‐led monitoring is one approach to grounding data collection in the real‐world experience of clients. Evaluating complex, context‐specific strategies to strengthen health outcomes in a way that is informative for other settings requires clear specification of the intervention packages that are planned and delivered in practice. Programme science provides a model for equitable partnership through co‐leadership between programmes, researchers and the communities they serve.

**Conclusions:**

Programme science addresses the FURRIE challenges, thereby improving programme impact and ultimately health outcomes and health equity. The adoption and adaptation of the types of novel programme science approaches showcased here should be promoted within and beyond the HIV/STI field.

## 1 INTRODUCTION

Programme science aims to improve the design, implementation and impact of public health programmes through the systematic application of theoretical and empirical scientific knowledge [1, 2, 3, 4, 5]. It is a framework for both programme implementation and research, defined by an iterative process whereby empirical and situated knowledge derived from programmes drives scientific inquiry, which then produces further evidence that is incorporated into programming for service optimization and population‐level impact and equity. Importantly, a programme science approach situates the scientific and operational leadership of research activities within, in part or in total, service delivery organizations. Integrating the administration of science within programmes promotes close coordination and alignment of objectives.

Delivering programme science requires addressing a range of practical and methodological challenges. These challenges are not unique to programme science but overlap with challenges faced in applied public health disciplines including implementation research [6], programme monitoring, pragmatic research [7], evaluations of complex interventions [8, 9] and quasi‐experimental studies [10], as well as encountering the broader ethical and practical challenges of undertaking research within resource‐constrained health systems.

Building on previous work [11], we have identified six challenges—identified by the acronym “FURRIE”—that programme scientists are developing ways to overcome. First, there are challenges in making studies within programme contexts *feasible* to undertake (note, the feasibility of undertaking research, rather than of delivering interventions). Once operationalized, research nested within programmes must be *useful*, providing actionable results for relevant stakeholders that consider how programmes are delivered, and the mechanisms by which they work and contextual factors that affect them. Programme science seeks to overcome the interdependent challenges of being scientifically *rigorous* (i.e. internally valid and providing unbiased answers), but also providing answers that are relevant to the *real‐world* (i.e. study conditions should, as much as possible, emulate conditions outside the research context). Research conducted within programmes must be *informative*, facilitating replication of successful programme strategies, supporting knowledge transfer and providing findings that can be included in evidence synthesis. Finally, there is growing attention to developing *equitable* partnerships that mitigate against the power imbalances that exist within the global health ecosystem [12].

Addressing all six challenges is critical to delivering programme science. This Debate paper discusses how the application of methodologies and approaches from applied public health research disciplines can help operationalize programme science by overcoming the “FURRIE” challenges. It does not seek to be exhaustive: we have not systematically reviewed the literature, nor widely consulted across the field. Rather, through face‐to‐face and online meetings, our group discussed the FURRIE challenges and identified examples from our own work that illustrate how these have been confronted. As such, the paper discusses a purposively selected set of cases illustrating the FURRIE challenges in programme science settings. It summarizes the context of each application, the FURRIE challenge addressed, the methodology or approach applied, and, reflects on the strengths, weaknesses and learning.

## 2 DISCUSSION

### 2.1 Increasing the feasibility of programme science

Feasibility challenges that programme scientists overcome include reconciling programme and research timelines, engaging stakeholders to ensure both independence and buy‐in, confronting the particular ethical issues of programme science, and reducing the costs of, and securing resource streams for, programme science. In general, the examples were made feasible by researchers, partly supported by research funding, working with programmes supported by implementation funds from multiple sources.

One way in which programme science can overcome feasibility challenges is through strengthening the analysis and interpretation of data collected in service delivery, sometimes referred to as “routine data.” Routine data hold the potential to provide a low‐cost, high‐volume data stream collected from clients, and/or potential clients, of programmes, with the potential to provide insights to drive programme improvement. However, careful handling, analysis, augmentation and interpretation are necessary [13].

One example of augmented routine data comes from India, as part of the Avahan‐Sankalp initiative which integrated violence‐prevention interventions for key populations with HIV prevention programming [14]. This example shows how consultation with female sex workers showed how violence was such an integral part of their lives that without addressing violence the programme could not achieve other outcomes. The routine data system was enhanced to capture experiences of violence against clients and the support that they received from the programme. Data review helped to refine the interventions delivered and linked to by the programme. For example, the data suggested the need for a shift from a focus on police violence to one on intimate partner violence. There were limitations: for example, although the reporting tools were visual, designed for use with peer educators, some of whom had limited literacy levels, regular training was needed. Outreach workers met weekly with peer educators to assess data quality. The work had dedicated programme science funding, but longer‐term funding supported the development of enhanced data collection, data extraction using visual tools for peers, data use and interpretation and application by peers and the programme.

In general, the expanded use of routine data is a necessary, but not sufficient, pillar to support programme science. Capacity to analyse, interpret and disseminate findings from routine data are essential. In addition, there is a middle ground between bespoke research data and routine programme data. For some questions, it is necessary to collect supplemental information to assess how far off the programme data are from reality. Measurement of a sample of observations has proved better than analytic approaches to address misclassification or missing data, which are based on assumptions that are rarely met.

For example, the Centre for Infectious Disease Research in Zambia (CIDRZ), with funding from the Bill & Melinda Gates Foundation (BMGF), investigated the outcomes of patients “lost to follow up (LTFU)” in the PEPFAR‐funded HIV treatment programme in Zambia. LTFU was defined as 90 days or more late from their last scheduled appointment. Using data from the national electronic medical record (EMR), a cohort of patients across four provinces was enumerated, with a random sample of patients LTFU traced by peer health workers to document their true outcome using a structured form. Using EMR data only, 42.7% (95% CI: 38.0−47.1%) of patients newly starting treatment between August 2013 and July 2015 were retained in care at 24 months, while 55.5% were lost and 2.2% had died. Using revised Aalen‐Johansen estimates incorporating tracing outcomes of LTFU patients [15], we found 77.3% (95% CI: 70.5−84.0%) retained at 24 months, 9.6% lost (95% CI: 8.7−10.5%) and 13.1% had died (95% CI: 12.2−14.1%). For this cohort, the use of routine programme data underestimated programme retention. The sampling‐based approach, by identifying a numerically small but randomly selected sample of participants, achieved a more realistic estimate of mortality among those who start antiretroviral therapy (ART), but for whom retention or mortality was not recorded in routine programme records. The limitations of this work were an imperfect response rate and the use of self‐reported care status. These data have been critical for driving programme improvement [16] and have been replicated with similar results using routine monitoring and evaluation tools in the context of the national HIV treatment programme [17].

### 2.2 Making programme science useful

Maximizing usefulness is at the heart of programme science. Programme science addresses questions of importance to programmes, stakeholders and beneficiaries and provides actionable answers to those questions that can be used by programmes. Process evaluation is one way to enhance the usefulness of programme science efforts, by forcing a focus on implementation processes, programme−client interactions and contextual factors.

In Zimbabwe, the Centre for Sexual Health and HIV/AIDS Research (CeSHHAR) undertook a process evaluation of a human‐centred design (HCD) intervention to promote voluntary medical male circumcision among adult males [18]. Interpersonal communication (IPC) agents who delivered the intervention were observed conducting sessions. Data collection included 24 in‐depth interviews with IPC agents and 5 with supervisors, and 8 focus group discussions with clients and 4 with IPC agents. Interventions were not delivered as intended. IPC agents found that providing targeted information to men individually, rather than providing more generic information to groups, resulted in men getting better information. However, they were able to reach (far) fewer men using HCD. IPC agents had targets to reach and performance management incentives made it more advantageous to target groups of younger boys in school settings than individual older men. These results suggested actionable implementation problems were an important explanatory factor in why the programme appeared to have had limited impact. Critically, the deviations from the intended implementation, and the reasons for it, were not visible to the implementers assessing target‐level data.

In Kenya, process evaluation provided important insights into self‐testing which were used to refine the programme, demonstrating the flexibility of the approach [19]. During 12 months of implementation, the programme science team conducted monthly meetings with programme teams to review monitoring data, assess progress in distribution, understand barriers to distribution and develop strategies to address the challenges. In‐depth interviews, rapid ethnographic assessments and polling booth surveys were deployed to understand programme coverage and user experience [20]. Based on the findings, new community‐based distribution channels were added. Subsequently, the programme increased its intensity to reach non‐testers or infrequent testers. This process monitoring also helped in designing and scaling up demand generation activities for men who have sex with men who met partners in virtual spaces.

The usefulness of programme science can also be enhanced by assessing how effects occur. Traditionally, pragmatism—simplicity and parsimony in measurement—has been equated with generalizability. However, the need to infer from a study into diverse real‐world settings means that even without study artefacts, studies in one programme setting may or may not enable inferences in another setting. Understanding the mechanisms of effects can help. Consider a quality improvement (QI) programme that improved performance by 50%, but also found that the effect depended heavily on healthcare worker acceptability of facilitators. If one of the major drivers of acceptability is leadership buy‐in, and another is organizational slack, then this insight could tell programme implementers where the QI programme is likely to have stronger and weaker effects on performance. In certain circumstances, additional measurement may extend, rather than undermine, external validities.

### 2.3 Keeping programme science rigorous

Programme science must strive for scientific rigour. In common with all intervention studies, this requires careful attention to issues of random error (statistical power and precision), selection bias, measurement error and confounding. While randomization does not in itself ensure the rigour of outcome evaluations, it is one powerful approach to limiting the effect of confounding. However, the “real‐world” context can render it challenging to undertake randomized evaluations of programme improvements, or may limit rigour through constraints to other aspects of study design.

We have successfully used cluster randomization in a range of studies, but, where this has not proved feasible, non‐randomized designs can be used. For example, in Lusaka, Zambia, the CIDRZ‐supported HIV programme sought, with funding from BMGF and the U.S. Centers for Disease Control and Prevention, to address patient disengagement from care by implementing differentiated service delivery (DSD) models to lower barriers to service access. The Fast Track (FT) model was implemented in 2017 for people living with HIV on ART ≥ 6 months, with WHO stage I/II disease and CD4 count >200 or virally suppressed. The model provided expedited clinical services that included a dedicated clinician and pharmacy team, designated space within the health facility and ART dispensation refills of ≥3 months. A retrospective cohort review covering 2 years of implementation in 14 high‐volume facilities (at least 3000 patients per month) showed that retention in care and viral suppression was superior among patients receiving care through the FT than those in standard of care (SOC). Of the 3284 patients in the FT model compared to 83,764 in SOC, 89.5% were retained at 12 months compared to 60.5%, respectively (*p*<0.001), while viral suppression was 95% compared to 89.1%, respectively (*p*<0.001) [21]. Missingness in the routine record hindered our ability to adjust for unmeasured confounders in the final analysis. This limitation notwithstanding, this DSD model is now part of the national HIV treatment guidelines and is implemented at scale across all districts in the country.

In Zimbabwe, as part of the HIV Self Testing Africa initiative, a non‐randomized difference‐in‐difference analysis was undertaken comparing communities with access to self‐testing and surrounding communities within the same districts without HIV self‐testing (HIVST) access. Ministry of Health programme data were used to compare facility‐based ART initiations related to self‐test distribution [22, 23]. Monthly ART initiations at public clinics were compared between 40 clinics with, and 124 without, HIVST distribution within their catchment area for three time periods (6 months prior to HIVST distribution; during the 6‐week HIVST campaign; and 3 months after the HIVST campaign). In clinic catchment areas included in the analysis, 12,808 ART initiations occurred, with no baseline or post‐campaign differences between initiation rates in HIVST versus non‐HIVST clinics. However, initiation rates increased from 7.31 to 9.59 initiations per month in HIVST clinics during the HIVST distribution campaign, aRR: 1.27, 95% CI 1.17−1.39, leading to the conclusion that community‐based HIVST campaigns achieved high testing uptake and were temporally associated with increased demand for ART. 

### 2.4 Keeping programme science relevant to the real‐world

Some research methodologies, especially those imported from clinical studies and/or other forms of explanatory human subjects research, can inadvertently undermine the “real‐world” nature of the settings in which they are deployed. Examples include where study participants are highly selected, perhaps because of their willingness to enrol in cohorts, and become unlike the source population that is the target for programme efforts. In other settings, intensive research consent procedures can act as a selection barrier to programmes operating as they would in real life. Research‐inspired intensive monitoring of programmes in ways that would not be replicable at scale offers a further example of how studies might provide data inadvertently de‐linked from appropriate real‐world inference. Programme science seeks to avoid these limitations.

One rapidly developing area that can overcome some of the limitations of a more formalized research process, but provide critical relevant insights to support programme improvement is community‐led monitoring (CLM) [24, 25]. Examples include health facility committees, client report cards, community scoring and community observatories. CLM can stay relevant by identifying early markers that programmes should monitor. For example, across a range of settings, the International Treatment Preparedness Coalition collected data on multi‐month dispensing of ART in September 2020 because it was particularly relevant to people living with HIV in the context of COVID‐19. A year and a half later, in February 2022, UNAIDS added multi‐month ART dispensing as an indicator in Global AIDS Monitoring. Work in Sierra Leone and Kenya emphasized the importance to clients, not of viral load testing being undertaken, but of having the result explained to them [25]. In South Africa and Malawi, CLM documented not only a COVID‐19‐induced drop in HIV testing access among female sex workers, but also identified barriers to testing which ultimately led to mitigation measures and a tripling of pre‐COVID testing rates. In each case, CLM allowed the collection, analysis and feedback of insights from data in a more nimble and responsive manner than a traditional research process, allowing insights to be translated more quickly into benefits for recipients of care. This demonstration work was funded directly, with the aim to disseminate widely and foster a wider take up on the approach.

More generally, although it has limitations, the PRECIS‐2 tool can help programme scientists consider whether their research efforts may be closer to or further from the “real world” settings in which they work [25]. Developed initially as a “pragmatic‐explanatory” continuum indicator applied mostly to drug trials, the tool highlights domains in which research designs can become less “pragmatic.” These include in relation to eligibility and recruitment of participants, and flexibility in delivery, monitoring of adherence or intensiveness of follow‐up procedures. In general, programme science studies seek to align delivery standards with the real world as much as possible. Our main aim in relation to study design is to maintain rigour (as described above), without imposing aspects of the research process that can de‐link the setting of research from the conditions of real‐world implementation.

### 2.5 Making programme science informative

It can be complex to describe exactly what a programme improvement intervention consisted of, and more complex still to consider whether a programme studied in one setting would be feasible to implement, achieve similar results or what adaptations it might require in other settings. While this problem has a range of dimensions, one area where there has been recent guidance that is useful for programme science relates to the development of standardized frameworks for capturing the specifics of what a complex intervention, or implementation strategy, consists of.

In the AMETHIST study in Zimbabwe [26], the TIDieR and Proctor frameworks were adapted to provide a structured view of the implementation components of our complex intervention combining peer‐led microplanning for community mobilization and self‐help groups [27, 28]. A brief description of the theoretical rationale for the intervention was provided, outlining who was planned to deliver the intervention components, and with what materials and procedures, as well as details of how, where and when the intervention should be delivered (all from TIDieR). The target client groups and the target client behaviours the interventions were intended to support were outlined (from Proctor), as were tailoring of the intervention for different client groups a priori. The final paper described issues of fidelity and, in particular, modification in the face of COVID‐19. Finally, the same framework was further adapted to briefly describe the existing platform on which the new interventions were overlaid, and which formed the services available in the comparison communities within our study. Through using these frameworks, the study reports are informative not only to our own setting but also to others considering implementing or evaluating these components of our interventions in other settings.

### 2.6 Equitable partnerships for programme science

Programme science has equity at its heart, and is well‐placed to operationalize equitable partnership models for co‐leadership among those who deliver, oversee, use and conduct research within programmes. As the field grows, programme science must monitor its efforts to live up to these ideals. Further, as suggested by both the inverse care law [29, 30] and inverse equity hypothesis [31], programme strengthening efforts, and the introduction of new innovations, can, paradoxically, exacerbate rather than improve health inequalities. Programme science must be attentive to these dynamics in contributing to the sustainable agenda for health.

Discussion of these issues is beyond the scope of this paper, as is the discussion of how issues of gender power are central to both the implementation and research challenges faced by programme science. We recognize these limitations. Nevertheless, across the examples cited above, programme science priorities were set in partnership with communities and implementers, and solutions developed with community‐led organizations. CLM proposes a paradigm shift in traditional power dynamics with communities leading the collection and analysis of the data, and recipients of care becoming data experts, rather than just data sources. Programme science offers a promising equitable partnership model that strives to ensure that it works to limit, rather than exacerbate, inequities in health access and outcomes.

## 3 CONCLUSIONS

Research in close partnership with STI/HIV programme delivery efforts is often conducted with marginalized populations, and in the context of powerful gender inequality dynamics. Programme science seeks to strengthen programme impact and improve health outcomes and health equity. There are unique but overlapping challenges in these contexts, characterized in this paper as the “FURRIE” challenges—relating to the feasibility, utility, rigour, real‐world‐ness and informative‐ness of programme science, and the equitable partnership models deployed to deliver it. The discussion draws on examples from the HIV/STI field, but the programme science and FURRIE challenges have relevance across the wider global health field.

The paper has reflected on some selected successes in deploying methods and approaches described in the scientific literature that overcome some of the FURRIE challenges. The paper aims to articulate the challenges, and shows a few examples of these being addressed, rather than to be exhaustive. We have illustrated some of the opportunities in applying these approaches, and hope this will be a useful resource for others planning and delivering programme science of the highest quality.

There are limitations in the approaches described. Partnerships between researchers and programme implementers require trust. It is challenging for implementers, who may be under pressure to meet delivery targets, to make the space for investigations of what they do. Those who fund programmes should reward implementers for doing more in‐depth investigation of issues, and resource these efforts appropriately. In addition, the tools and language of research sometimes act as barriers. Researchers must dedicate significant effort to being guided by programme science principles, being aware of the FURRIE challenges. Where these are addressed, “science” can be applied, usefully and with impact, in real‐world programme settings, and as such, programme science offers an approach to leveraging the power of science for change.

## COMPETING INTERESTS

The authors declare no competing interests.

## AUTHORS’ CONTRIBUTIONS

JH conceptualized the manuscript and drafted the initial text. SB, PB, FMC, MH, KL, IS and EG provided details of specific programme science examples from their own work and made significant edits to the text. All authors read and approved the final manuscript.

## Data Availability

Data are available on request from the authors.
